# Artificial intelligence in biomedical team science: perceptions, practices, and training needs

**DOI:** 10.3389/fpsyg.2025.1720970

**Published:** 2026-01-21

**Authors:** Emily Slade, Kelsey Karnik, Caitline Phan, Megan E. Hall, Yana Feygin, Kristen J. McQuerry

**Affiliations:** Department of Biostatistics, University of Kentucky, Lexington, KY, United States

**Keywords:** artificial intelligence, biomedical research, collaboration, team processes, team science

## Abstract

**Introduction:**

Artificial intelligence (AI) is increasingly used in biomedical research, yet limited empirical work has described how researchers use AI tools on collaborative research teams and how they view their role within team-based research. This study examines researchers’ experience with and attitudes toward AI use in collaborative research environments.

**Methods:**

A cross-sectional survey was administered to 178 investigators engaged in collaborative research at the University of Kentucky. Questions assessed AI use across research and communication tasks, team-related decision-making practices, perceived benefits and concerns, and preferences for training and frameworks.

**Results:**

Thirty-nine participants responded (22%). AI use was heterogeneous: 26% had never used AI on research teams, while 42% used it weekly or daily. Nearly half reported that AI use was not discussed within teams prior to starting the work. Participants identified benefits in reducing repetitive tasks but expressed widespread concerns about misinformation, bias, and overreliance. Most participants indicated interest in self-guided training and structured frameworks, with priority topics including data security, ethical use, and practical strategies for team integration.

**Discussion:**

Findings indicate variability in both the use of AI tools on research teams and researchers’ attitudes toward their integration. Results highlight gaps between perceived benefits and current practices and suggest a need for evidence-based training and frameworks that support responsible and effective AI use on collaborative research teams.

## Introduction

While the use of artificial intelligence (AI) in biomedical research is rapidly expanding, most existing literature has emphasized technical performance and applications rather than the social and collaborative dimensions of AI integration within research teams. Emerging work in the science-of-team-science literature has begun to explore how digital tools shape coordination, communication, and decision-making; however, few studies have examined how AI is understood, discussed, or adopted in multidisciplinary research settings.

Recent studies have examined how individual scientists are engaging with AI tools. One large, international survey of researchers found that many were using AI for tasks such as coding, manuscript drafting, literature exploration, and data handling, and a substantial proportion viewed these tools as potentially important for the future of their fields (Van Noorden and Perkel, 2023). Despite this interest, respondents expressed significant concerns about misinformation, plagiarism, bias, reproducibility, and threats to scientific integrity, underscoring the need for clearer norms and safeguards around AI use. A separate, U.S.-based survey of academic researchers reported similarly cautious engagement: while many respondents indicated curiosity about generative AI and used it for tasks like summarization, editing, and idea exploration, far fewer reported using it for core scientific activities such as hypothesis generation or analytic work (Ruediger et al., 2024). Similar findings have been reported among biostatisticians using large language models (Grambow et al., 2025). These researchers reported commonly using AI to improve productivity in coding, writing, and literature review, but they also reported frequently encountering error-prone outputs. They also expressed strong interest in structured training, case studies, and verification strategies to support responsible and effective use. Although these surveys provide important insights into individual use, they do not capture how AI use is coordinated, discussed, or negotiated within research teams, leaving a critical gap in understanding the social dynamics of AI integration on research teams.

Given this gap, it is important to recognize that the effects of AI on teams are unlikely to be uniform. How teams adopt and use AI can vary widely depending on individual differences among team members, such as prior experience with digital tools, openness to innovation, and disciplinary background (Man Tang et al., 2022; Wang et al., 2024), the design and functionality of AI tools themselves (Harris-Watson et al., 2023), and the strategies used to implement AI within collaborative workflows (Bezrukova and Griffith, 2025; Schelble et al., 2024; Harris-Watson et al., 2023). These factors interact in complex ways and may support or hinder team coordination, communication, and trust.

This variability aligns with key concepts from established and emerging frameworks for understanding team science. The team mental models (TMM) framework emphasizes that effective teamwork depends on a shared, organized understanding of roles, tools, and workflows (Cannon-Bowers et al., 1993; Klimoski and Mohammed, 1994). Several domains assessed in the present survey (e.g., clarity surrounding responsibility for AI-related decisions, communication about how AI should be used) reflect elements central to TMM theory. Complementing this perspective, recent conceptual work on AI-specific team integration highlights how team attitudes toward AI, as well as whether its use is voluntary or mandated, shape collaboration dynamics and adoption strategies (Bezrukova et al., 2023). Together, these frameworks provide a lens for considering both general and AI-specific factors that influence how teams incorporate AI into research workflows.

Existing empirical work on AI and collaboration underscores the importance of examining how teams incorporate these tools into their work. Emerging evidence suggests that when AI systems perform well, they may reduce perceived conflict among team members (Dennis et al., 2023) and free members from repetitive tasks, creating more opportunities for creative problem-solving and higher-order research activities (Dell’Acqua et al., 2023). Conversely, other studies caution that AI adoption may introduce process losses or undermine critical thinking and decision-making (Demir et al., 2017; Schmutz et al., 2024; Vaccaro et al., 2024). Together, this mixed evidence highlights the need for empirical studies that examine not only whether AI is used, but also how teams make decisions about its role and the specific research- and teamwork-related tasks for which it is (or is not) incorporated.

Building on these theoretical and practical considerations, this study investigates how researchers across biomedical disciplines perceive and experience the integration of AI tools in collaborative, multidisciplinary research. Using a structured survey administered to investigators at the University of Kentucky, we examine attitudes about the use of AI on research teams, the extent to which AI is currently being used across research and communication workflows, and who leads decisions about its use. The survey also assesses perceived risks, desired training, and interest in structured frameworks to guide ethical and effective AI integration. By capturing researchers’ perspectives across multidisciplinary teams, this study provides initial, real-world insights that can help inform the development of guidance and frameworks to support responsible and effective use of AI on collaborative research teams.

## Methods

This study was a cross-sectional survey of investigators engaged in multidisciplinary research at the University of Kentucky. Data were collected using a self-administered survey developed and distributed through REDCap (Harris et al., 2009). The survey included items on demographics and professional background (e.g., role on research teams, primary research domain, number of recent collaborations), experiences with and use of AI tools in collaborative research, perceptions of risks and benefits, and preferences for decision-making, training, and frameworks to support responsible AI use on multidisciplinary research teams. For the purposes of the survey, an AI tool was defined as “a software application that uses artificial intelligence techniques, such as machine learning or natural language processing, to perform tasks that typically require human intelligence.” The full survey is provided in Supplementary material. The study was approved by the University of Kentucky Institutional Review Board (#105228).

Investigators were eligible to participate if they initiated a new collaboration with the institution’s collaborative biostatistics core between July 1, 2023, and June 30, 2025, had a valid institutional email address, and were actively affiliated with the institution at the time of survey launch (August 20, 2025). Of 193 investigators meeting the collaboration window, 11 were excluded due to inactive or invalid email addresses, and 4 were excluded because automated messages indicated they were no longer employed at the University, yielding a final sample of 178 eligible participants.

Because investigators seeking biostatistical collaboration are, by definition, engaged in at least one multidisciplinary, team-based research study, this population provided a practical proxy for researchers working in collaborative contexts. Surveys were distributed to investigators as individuals rather than to entire teams, which allowed us to capture each participant’s perspectives across all of the collaborative teams they are involved in, rather than being limited to the dynamics of a single team. Three reminder emails were sent approximately weekly, and the survey remained open for a total of 4 weeks.

Survey responses were analyzed descriptively, with categorical variables summarized as frequencies and percentages. All analyses were performed in R version 4.2.0. Likert scale responses were summarized graphically using the *gglikert()* function within the ggstats package (Larmarange, 2023).

## Results

Of 178 eligible participants, 39 (21.9%) responded to the survey. Among respondents, 23.1% were 25–34 years old, 41.0% were 35–44 years old, 23.1% were 45–54 years old, and 12.8% were 55 or older. Gender was approximately balanced between females (55.3%) and males (44.7%). The majority of participants identified as White (84.2%) and not of Hispanic or Latino/a origin (92.1%). Table 1 includes complete information on participant characteristics.

**TABLE 1:** Participant demographics, research experience, and AI use.

Characteristic	*N* (%)
Demographics	
Age	
25–34 years	9 (23.1%)
35–44 years	16 (41.0%)
45–54 years	9 (23.1%)
55–64 years	4 (10.3%)
65 years or older	1 (2.6%)
Gender identity	
Man	17 (44.7%)
Woman	21 (55.3%)
Missing	1
Race	
Asian	2 (5.3%)
Black or African American	2 (5.3%)
White	32 (84.2%)
Two or more races	2 (5.3%)
Missing	1
Ethnicity	
Hispanic or Latino/a origin	3 (7.9%)
Not of Hispanic or Latino/a origin	35 (92.1%)
Missing	1
Research team characteristics	
Typical role on research teams	
Investigator (e.g., PI, Co-Investigator)	34 (87.2%)
Professional research support staff	2 (5.1%)
Trainee (e.g., student, post-doc)	3 (7.7%)
Primary research domain	
Basic science	3 (7.7%)
Clinical research	16 (41.1%)
Data/quantitative science	7 (17.9%)
Education	1 (2.6%)
Public health/community-engaged research	8 (20.5%)
Social/behavioral science	1 (2.6%)
Translational science	3 (7.7%)
Number of research teams you were a member of in the past 2 years	
1	2 (5.1%)
2–5	23 (59.0%)
6–10	10 (25.6%)
More than 10	4 (10.3%)
AI use and training	
How often do you use AI tools in your work on collaborative research teams?	
Daily	2 (5.3%)
Weekly	14 (36.8%)
Monthly	6 (15.8%)
Rarely (a few times per year or less)	6 (15.8%)
Never	10 (26.3%)
Missing	1
Have you received any training or guidance on using AI tools in the collaborative research environment? (select all that apply)	
No	24 (61.5%)
Yes, informal training (e.g., self-directed	14 (35.9%)
learning)	
Yes, formal training (e.g., workshops, courses)	1 (2.6%)
Not sure/don’t recall	1 (2.6%)

Percentages are calculated among those who responded to each survey question. Missing data in each question is indicated; any variables without a “Missing” row did not have any missing data.

When asked about their typical role on collaborative research teams, 34 (87.2%) indicated that they were an investigator (e.g., PI, Co-Investigator), 2 (5.1%) indicated that they were professional research support staff (e.g., project director, lab technician), and 3 (7.7%) indicated that they were a trainee (e.g., student, post-doc). Primary research domains of these participants included clinical research (41.0%), public health/community-engaged research (20.5%), data/quantitative science (17.9%), translational science (7.7%), basic science (7.7%), social/behavioral science (2.6%), and education (2.6%). Most participants (59.0%) indicated that they have participated in approximately 2–5 collaborative research teams in the past 2 years, with smaller numbers indicating that they participated in 1 collaborative research team (5.1%), 6–10 collaborative research teams (25.6%), or more than 10 collaborative research teams (10.3%).

### Individual AI use on research teams

Participants’ individual use of AI tools on collaborative research teams was mixed. About one-quarter (26.3%) had never used an AI tool to support any aspect of their work on collaborative research teams, 31.6% used AI tools rarely or monthly, 36.8% used AI tools weekly, and 5.3% used AI tools daily (Table 1). Of the 28 participants who used AI tools to support their work on collaborative research teams, most (96.4%) reported using ChatGPT. The next most commonly used AI tools were Microsoft Copilot, Google Gemini, and Grammarly, though only ≤25% of participants used each of these.

Participants who use AI tools to support their work on collaborative research teams (*n* = 28) were asked to indicate which tasks they have used AI tools for. Full results are shown in Table 2. Among research-related tasks, the task with the most AI use was “drafting or editing dissemination materials (e.g., manuscripts, grant proposals, abstracts)” (46.4%), followed closely by “generating or debugging code for statistical or computational analyses” (39.3%) and “conducting literature review or synthesizing background materials” (35.7%). Among communication and coordination-related tasks, the majority of participants (57.1%) used AI tools for “drafting or editing emails or messages.” AI use was less common for other communication and coordination-related tasks, with the next most frequent being “creating meeting agendas or planning documents” (25.0%) and “taking automated meeting notes” (21.4%).

**TABLE 2:** Use of AI tools in research tasks and communication/coordination tasks on research teams (*N* = 28).

Across any of your research collaborations, for which of the following research tasks have AI tools been used to support the work?	*N* (%)
Drafting or editing dissemination materials (e.g., manuscripts, grant proposals, abstracts)	13 (46.4%)
Generating or debugging code for statistical or computational analyses	11 (39.3%)
Conducting literature review or synthesizing background materials	10 (35.7%)
Preparing visual materials (e.g., slide decks, figures, data visualizations)	9 (32.1%)
Developing marketing or outreach materials (e.g., for participant recruitment)	7 (25.0%)
Interpreting outputs from statistical or computational analyses	6 (21.4%)
Designing studies or planning research projects	5 (17.9%)
Drafting or editing regulatory documents (e.g., IRBs, study protocols, DSMB charters)	5 (17.9%)
Other	4 (14.3%)
Running data analyses directly within an AI tool (e.g., uploading data into an AI interface)	1 (3.6%)
**Across any of your research collaborations, for which of the following communication or coordination tasks have AI tools been used?**	***N* (%)**
Drafting or editing emails or messages	16 (57.1%)
Creating meeting agendas or planning documents	7 (25.0%)
Taking automated meeting notes (e.g., via transcription tools)	6 (21.4%)
Summarizing or outlining meeting notes or discussions	3 (10.7%)
Mediating conflict or misunderstandings (e.g., drafting neutral responses, rephrasing feedback)	3 (10.7%)
Supporting onboarding of new team members (e.g., generating overviews, orientation materials)	2 (7.1%)
Clarifying team roles or responsibilities (e.g., drafting charters, identifying expertise gaps)	1 (3.6%)
Other	0 (0.0%)

Percentages represent the proportion of participants who indicated using an AI tool for the particular task, among those who have ever used an AI tool to support their work on a collaborative research team (*n* = 28). “Other” responses for research tasks included using AI tools to help guide Excel use for data management, re-phrasing writing, and providing theoretical information about a topic.

### AI and team processes

Participants reported on how often AI tool use is addressed within their collaborative research teams. Almost half (43.6%) of participants reported that across all of their collaborative research teams, discussions amongst team members about the use of AI tools have never occurred before beginning the work. Just over half (53.8%) reported that these discussions sometimes occur before beginning the work, and only one participant (2.6%) reported that they always occur. All but two participants (92.3%) agreed or strongly agreed that successful integration of AI tools into collaborative research workflows requires teams to discuss and develop clear internal policies about how AI will be used.

Participants’ opinions about who should be responsible for deciding how AI tools are used (or not used) within a research team were varied: 51.3% felt that the team should make this decision collectively though consensus or group agreement, 28.2% felt that the principal investigator or team leader should make the decision for the team, 10.3% felt that the institution or organization should establish guidelines that apply to all researchers in the organization, and 7.7% felt that each team member should decide independently how they use AI tools in their own work.

### Attitudes about AI use on research teams

**FIGURE 1:**
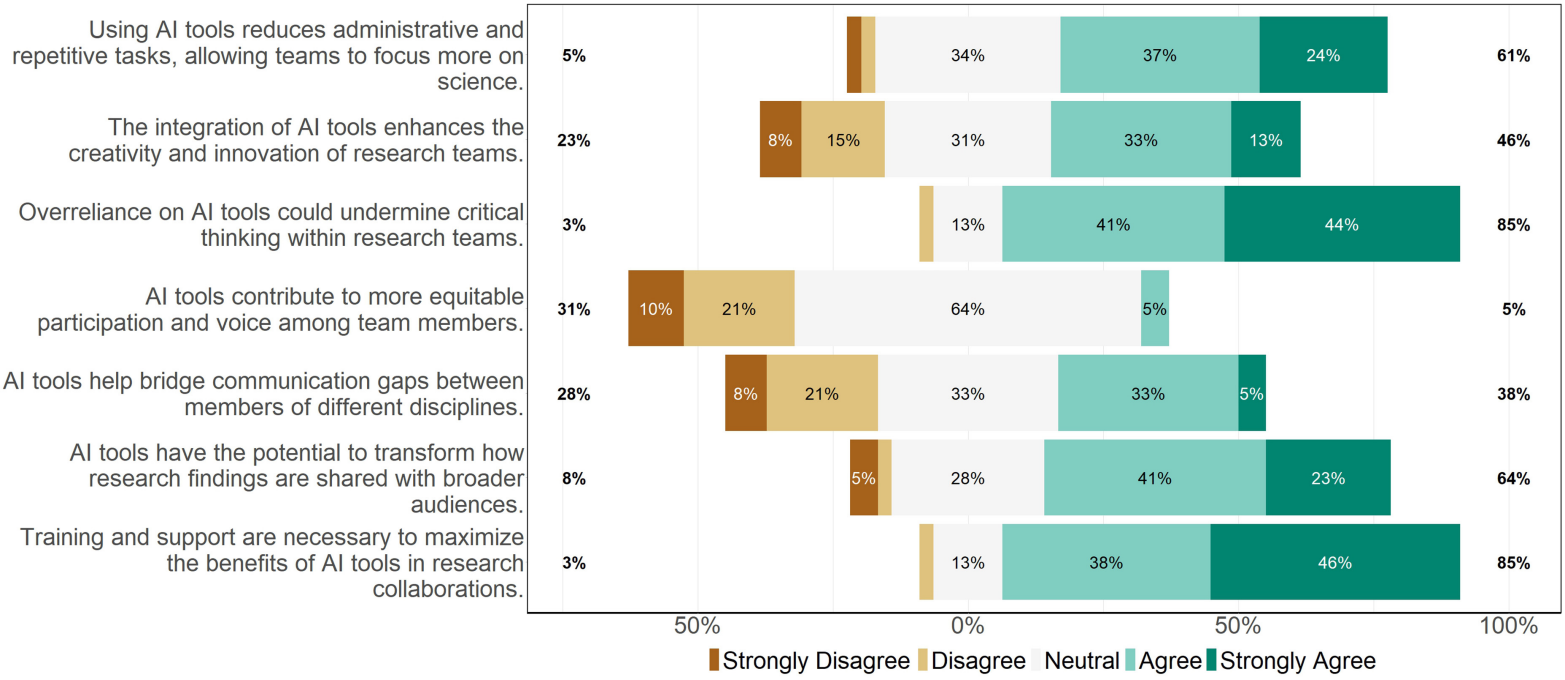
Individual attitudes about AI use on collaborative research teams (*N* = 39). Participants evaluated each statement on a 5-point Likert scale (“Strongly disagree,” “Disagree,” “Neutral,” “Agree,” “Strongly agree”). Percentages on the left side of the plot represent the proportion who selected “Disagree” or “Strongly disagree.” Percentages on the right side of the plot represent the proportion who selected “Agree” or “Strongly agree.”

Figure 1 shows responses to seven questions related to attitudes about AI use on research teams. The majority of participants (60.5%) agreed that using AI tools reduces administrative and repetitive tasks, allowing teams to focus more on science. However, most (84.6%) agreed that overreliance on AI tools could undermine critical thinking on research teams. There were mixed feelings about whether the integration of AI tools enhances the creativity and innovation of research teams: 46.1% agreed with this statement, 30.8% were neutral, and 23.1% disagreed. There were similarly mixed feelings about whether AI tools help bridge communication gaps between members of different disciplines: 38.4% agreed, 33.3% were neutral, and 28.2% disagreed. Most participants (64.1%) agreed that AI tools have the potential to transform how research findings are shared with broader audiences, and the vast majority (84.7%) agreed that training and support are necessary to maximize the benefits of AI tools in research collaborations.

All participants expressed at least one concern about using AI tools in collaborative research. Four concerns were particularly widespread, each expressed by over three-quarters of participants, including accuracy of outputs/misinformation (100.0%), misuse or overreliance on AI (89.7%), data/privacy concerns (79.5%), and bias in AI models (79.5%). Other concerns included intellectual property/plagiarism (66.7%), uncertainty about policies on AI use in collaborative research (56.4%), lack of transparency/black box (51.3%), environmental sustainability (41.0%), and job displacement (30.8%).

### Training and framework for AI use on research teams

In terms of training, 61.5% of participants had never received any training or guidance on using AI tools in the collaborative research environment. Of the 15 participants who had received training or guidance, the vast majority (93.3%) received informal training (e.g., self-directed learning, peer guidance, online tutorials) as opposed to formal training (e.g., workshops, courses, webinars) (Table 1).

**FIGURE 2:**
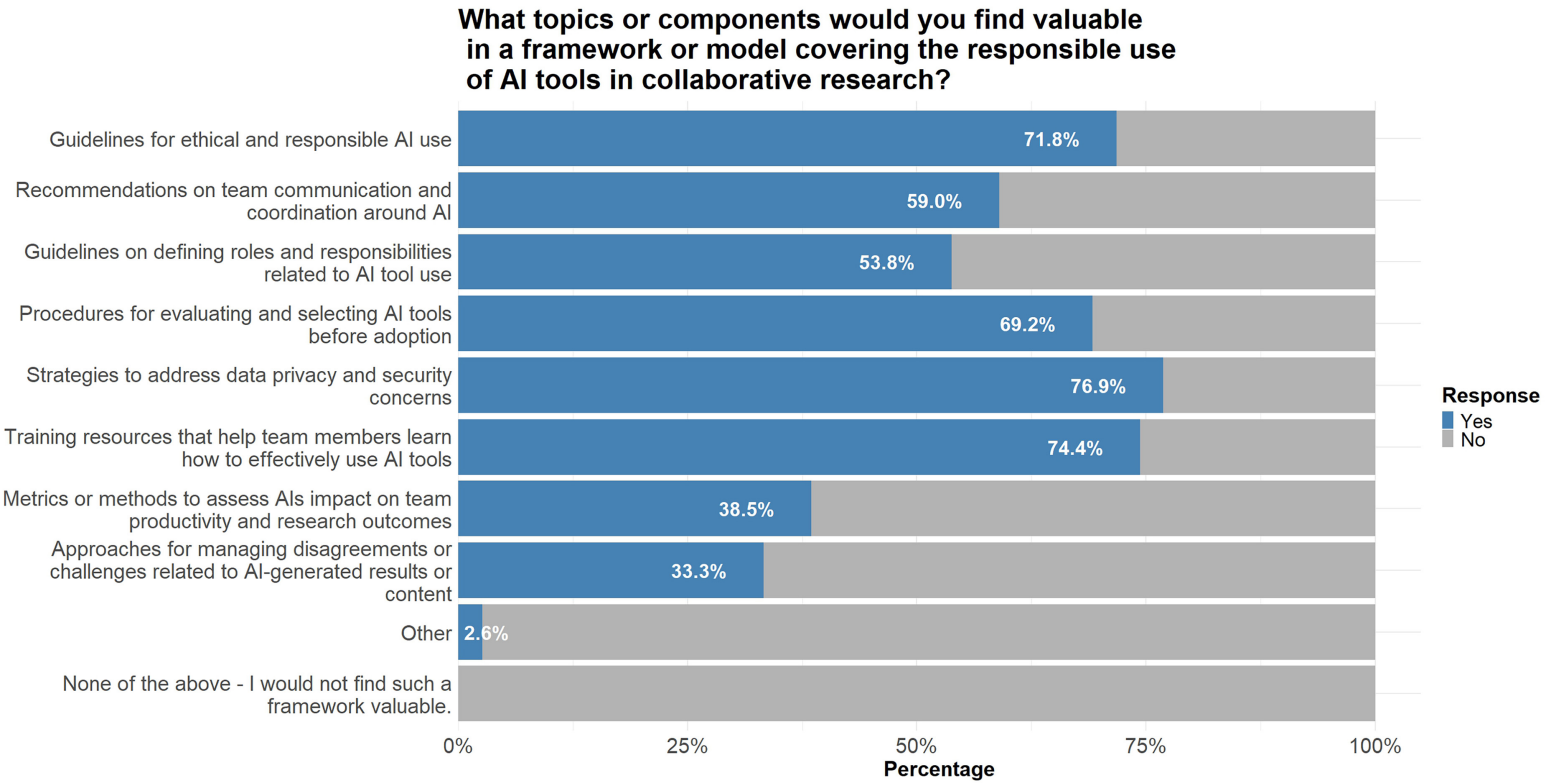
Participants’ opinions on topics or components that they would find valuable in a framework or model covering the responsible use of AI tools in collaborative research (*N* = 39). Percentages represent the proportion of participants who indicated that the given topic or component is something that they would find valuable in a framework or model covering the responsible use of AI tools in collaborative research.

Among all participants, 71.8% agreed or strongly agreed that they would benefit from a validated framework or model for the responsible use of AI in collaborative research. Figure 2 shows the topics or components that participants would find valuable in such a framework. The top three topics that the largest proportion of participants indicated they would find valuable in such a framework were: “strategies to address data privacy and security concerns” (76.9%), “training resources that help team members learn how to effectively use AI tools” (74.4%), and “guidelines for ethical and responsible AI use” (71.8%) (Figure 2). With respect to the format for receiving training or guidance on responsibly using AI in collaborative research, the majority indicated that they would prefer “online interactive courses or webinars” (66.7%) or “written guidelines or best practice documents” (59.0%). A substantial minority would prefer “in-person workshops or seminars” (43.6%), and smaller numbers would prefer “one-on-one coaching or consultation” (17.9%) or “peer discussion groups or communities of practice” (20.5%).

## Discussion

This study is one of the first to assess biomedical researchers’ attitudes toward, and adoption of, AI tools in collaborative research settings. Our findings highlight both enthusiasm and uncertainty. While many participants acknowledged potential benefits of AI, particularly in reducing administrative burden and supporting scientific tasks, actual use patterns and team-level practices reveal considerable variation and inconsistency. Notably, a substantial portion of participants reported limited or no engagement with AI tools (26.3% never used AI; 15.8% used it rarely). While detailed observations of specific AI use behaviors are therefore limited, documenting low or infrequent use is itself an important finding and demonstrates consistency with non-use rates of 22% and 37% reported in a 2023 and 2024 survey, respectively (Van Noorden and Perkel, 2023; Ruediger et al., 2024). Understanding when and why AI tools are not being used provides critical insight into current adoption patterns and highlights opportunities for guidance, training, and the development of shared team norms.

One striking result is the lack of clarity around responsibility for decision-making about AI use on research teams. Participants’ opinions were widely distributed, with some favoring collective decision-making, others deferring to principal investigators or institutional policies, and a smaller proportion endorsing individual discretion. This uncertainty is compounded by the fact that discussions about AI use within teams were infrequent, with nearly half of respondents reporting such conversations had never occurred in their collaborations. This pattern suggests variability in how leadership related to AI use is distributed within teams, consistent with concepts of shared or distributed leadership in collaborative work (Carson et al., 2007). In the absence of explicit norms, AI-related decisions may default to informal authority structures or individual discretion rather than being treated as a shared responsibility. Taken together, these findings suggest that many teams are integrating AI tools (or not) in an *ad hoc* fashion, without shared norms or explicit policies to guide responsible use.

Researchers reported a mix of optimism and concern regarding the use of AI tools on collaborative research teams. Most (60.5%) agreed that AI tools have potential to reduce repetitive tasks and create more space for scientific focus, a proportion notably higher than the 36.3% reported in a 2023 survey (Van Noorden and Perkel, 2023). However, widespread concerns about misinformation, bias, privacy, and overreliance reflect a strong undercurrent of caution, consistent with prior survey work (Van Noorden and Perkel, 2023; Ruediger et al., 2024). Importantly, these concerns may have implications for psychological safety–the shared belief that it is safe to speak up, question assumptions, or express uncertainty–within teams (Edmondson, 1999). Effective AI use often requires team members to question outputs, surface uncertainty, or challenge inappropriate reliance on automated tools; however, such behaviors depend on a climate in which individuals feel comfortable raising concerns or admitting limitations in their understanding of AI. If psychological safety is low, team members may be reluctant to question AI-generated outputs or to initiate conversations about appropriate use, potentially amplifying risks associated with overreliance or uncritical adoption.

Interestingly, while participants endorsed the potential for AI to save time on administrative work, actual reported use of AI for coordination-related tasks, such as drafting agendas or taking meeting notes, was relatively limited. It is possible that in some teams, these tasks are handled by other members who take advantage of AI, meaning that individual reports may underestimate team-level administrative use. AI use on teamwork-related tasks such as managing conflict, supporting onboarding of new team members, and clarifying team roles was also limited. Instead, the most common applications of AI involved research-facing tasks like drafting dissemination materials, coding, or synthesizing background literature. This discrepancy suggests that perceived benefits may not yet align with practice, perhaps because researchers lack tested tools, clear expectations, or confidence in applying AI in these domains.

Several findings extend and refine existing theoretical frameworks in ways that are particularly relevant for team science in the AI era. In particular, the observed inconsistencies in role clarity, limited team discussion of AI use, and lack of explicit decision-making structures suggest that key elements of the TMM framework (e.g., shared understandings of tools, workflows, and responsibilities) are underdeveloped in these teams. Viewed through this lens, AI tools represent an emerging component of teams’ working infrastructure that may be difficult for teams to integrate into shared mental models, given their adaptive nature and limited transparency. When shared understandings about AI are incomplete or misaligned, teams may struggle to coordinate their use of these tools, even when individual members recognize their potential benefits. Consistent with prior work demonstrating that greater convergence in team mental models supports more effective coordination and performance (DeChurch and Mesmer-Magnus, 2010), these findings suggest that misalignment around AI use may represent a meaningful source of coordination loss in collaborative research teams.

These challenges may be especially pronounced in multidisciplinary research teams, where boundary-spanning work is already required to integrate diverse forms of expertise, terminology, and epistemic norms (Stokols et al., 2008). AI tools may introduce an additional layer of complexity if they are unevenly understood or valued across disciplines, potentially exacerbating existing asymmetries in knowledge, power, or influence. In such contexts, unclear norms around AI use may hinder collaboration rather than support it, underscoring the importance of explicitly addressing AI as part of broader efforts to support interdisciplinary integration.

In parallel, the attitudinal patterns observed in this study (e.g., perceived usefulness of AI for reducing administrative workload, concerns about misinformation, varying levels of trust) align with established technology acceptance frameworks, which highlight that adoption depends not only on perceived utility but also on expectations, trust, and perceived barriers (Davis, 1989; Venkatesh et al., 2003). However, our findings also suggest a need to move beyond individual-level acceptance to account for team-level dynamics. Even when perceived usefulness of AI is high, adoption may remain inconsistent in the absence of shared norms, psychological safety, and clearly articulated leadership or governance structures. This highlights a team-level layer of technology acceptance that is under-theorized in existing models and helps explain why research-facing AI applications are more prevalent than teamwork-facing ones in collaborative research environments.

The heterogeneity in AI use observed in this study further underscores the importance of accessible guidance and training. While formal, synchronous training opportunities can be valuable, they are inherently limited in reach and scalability. For example, a recent workshop on AI in research hosted at our institution attracted 330 registrants but was limited to 80 seats. Survey participants expressed stronger interest in flexible, self-guided resources such as online interactive courses or written guidelines rather than formal, synchronous sessions. Prioritizing these adaptable formats could both align with researchers’ learning preferences and allow training to reach a wider audience, helping teams adopt AI responsibly even in the absence of extensive in-person instruction.

In addition to informing the modality of training, the patterns observed here offer preliminary insights that can inform the content of trainings and the design of actionable frameworks for responsible AI use in team-based research. Based on these findings, several recommendations emerge. First, trainings or guidance should consider promoting discussions about AI use early in the research process, such as during project kickoff meetings, to support psychological safety and the development of shared mental models. Second, teams or institutions may wish to consider explicitly clarifying decision-making authority and leadership roles related to AI use among team members. Third, training or frameworks focused on leveraging AI in team-based research may reasonably prioritize administrative and repetitive tasks as low-risk, high-value entry points for AI use (e.g., drafting agendas, taking notes, managing routine communications), as these applications may allow teams to realize benefits while minimizing concerns related to scientific integrity. Collectively, these elements could be incorporated into scalable training materials or practical frameworks for responsible AI use in team-based research (e.g., governance checklists, role-clarification templates, online training modules). Importantly, such trainings or frameworks should be treated as testable interventions rather than static guidance and evaluated empirically for their effects on team outcomes, including perceived role clarity, psychological safety, frequency and quality of team-level discussions about AI, and consistency of AI adoption across team members.

While these findings offer important insights for developing training and guidance, they should be interpreted in the context of study limitations. The survey achieved a response rate of 21.9%, which is relatively low and may limit the generalizability of the findings. Such response rates are not uncommon in broad, non-personalized email surveys of busy academic investigators, but the potential for nonresponse bias should be acknowledged. It is possible that respondents tended to be those who felt particularly strongly (either positive or negative) about AI use in collaborative research. Because we could not obtain demographic information on non-respondents, we were unable to assess potential selection bias. Given the limited sample size, subgroup analyses were not feasible in this pilot study, but examining differences across researcher roles, disciplines, and levels of AI use will be an important direction for future, larger-scale work.

Importantly, this study was designed as a pilot to provide preliminary insights into how AI tools are being integrated into research teams. In that context, the modest response rate is less problematic, as the primary aim was to inform the development of a framework for supporting team science, with future follow-up and more systematic evaluation anticipated before broader implementation. While this study did not measure the direct effects of AI on team functioning, communication, or role clarity, the observed patterns offer a foundation for subsequent research that can more directly test hypotheses derived from team science and technology adoption theories. For example, future larger-scale and multi-institution studies could examine whether interventions aimed at strengthening shared mental models, clarifying decision-making roles, or normalizing team-level discussions about AI lead to more consistent and responsible adoption across collaborative research teams. This study was conducted within a single large academic medical center, which offers relevance to similar institutions but may limit generalizability to settings with different team structures, resources, or institutional policies related to AI use in research. For broader inference across biomedical team science, the findings in this pilot study should therefore be used to guide the design of subsequent larger-scale and multi-institution studies that evaluate both the implementation strategies and team-level outcomes.

Another limitation of this study is the sampling approach. The survey was distributed to individual investigators who submitted requests for collaboration with our institution’s biostatistics core, rather than to entire, well-defined research teams. While this population serves as a practical proxy for researchers engaged in multidisciplinary work, the study does not capture complete team-level dynamics. As a result, the findings reflect individual perceptions and experiences of AI integration in team science, rather than a comprehensive, multi-perspective view from entire teams. Future research could benefit from a team-based sampling strategy to triangulate perspectives within and across teams to further assess how AI tools influence collective processes and outcomes.

Overall, our findings highlight both the urgency and the opportunity for structured approaches to AI use in team science. By situating AI adoption within established frameworks of team cognition and technology acceptance, and by identifying concrete leverage points for scalable training and governance, this study provides a foundation for translating AI enthusiasm into coordinated, responsible practice. As digital tools continue to evolve rapidly, models that explicitly integrate AI into the fabric of interdisciplinary collaboration will be essential for sustaining effective and adaptive biomedical research teams.

## Data Availability

The datasets presented in this article are not readily available because the University of Kentucky Institutional Review Board requires review prior to sharing data with researchers outside the study team. Requests to access the datasets should be directed to Emily Slade, emily.slade@uky.edu.
