# Supplementary material for: Artificial intelligence in biomedical team science: perceptions, practices, and training needs
Source: Front Psychol. 2026 Jan 21;16:1720970. doi: 10.3389/fpsyg.2025.1720970 (PMC12867778; doi:10.3389/fpsyg.2025.1720970)
Supplement: Supplementary file 1 [file Data_Sheet_1.pdf]

AI Use on Research Teams: Survey Questions

This study was approved by the University of Kentucky Institutional Review Board (#105228).

<u>Demographics</u>

Please select your age category.

- 18-24 years
- 25-34 years
- 35-44 years
- 45-54 years
- 55-64 years
- 65 years or older
- Prefer not to respond

Please indicate your gender identity.

- Woman
- Man
- Transgender Woman
- Transgender Man
- Non-binary/Gender Fluid
- Prefer to self-describe [*free text*]
- Prefer not to respond

Please indicate your racial identity.
Select all that apply.

- American Indian or Alaskan Native
- Asian
- Black or African American
- Native Hawaiian or Other Pacific Islander
- White
- Prefer to self-describe [*free text*]
- Prefer not to respond

Please indicate your ethnic identity.

- Not of Hispanic or Latino/a origin
- Hispanic or Latino/a origin
- Prefer to self-describe [*free text*]
- Prefer not to respond

<u>Research Role/Background</u>

Throughout this survey, "collaborative research teams" refer to multiple researchers working together to answer a common research question.

What is your typical role on collaborative research teams?

- Investigator (e.g., PI, Co-PI, Co-Investigator, Affiliated Faculty, Research Scientist)
- Professional Research Support Staff (e.g., Project Director; Research Associate; Research, Lab, Data, or Web Technician)
- Student or Trainee (e.g., Pre-Doc, Post-Doc, Student)
- Other [*free text*]

Which of these would you consider your <u>primary</u> research domain?

- Clinical Research (e.g., patient-oriented research, clinical trials, outcomes research)
- Basic Science (e.g., molecular biology, genetics, biochemistry, lab-based research)
- Data/Quantitative Science (e.g., biostatistics, bioinformatics, AI/ML)
- Public Health/Community-Engaged Research (e.g., epidemiology, health promotion, implementation science, community-based participatory research)
- Translational Science (e.g., bridging basic science and clinical practice, T1-T4 research)
- Social/Behavioral Science (e.g., psychology, sociology, human factors, organizational behavior)
- Other [*free text*]

Approximately how many collaborative research teams have you participated in during the last 2 years?

- 1
- 2-5
- 6-10
- More than 10

Actual Use of AI in Team Research

In this survey, an AI tool refers to a software application that uses artificial intelligence techniques—like machine learning or natural language processing—to perform tasks that typically require human intelligence.

These tools, such as ChatGPT, Microsoft Copilot, and more, may be used to recognize patterns, generate content, or assist with complex problem-solving.

Have you received any training or guidance on using AI tools in the collaborative research environment?
[First two options can be selected together]

- Yes, formal training (e.g., workshops, courses, webinars)
- Yes, informal training (e.g., self-directed learning, peer guidance, online tutorials)
- No
- Not sure/Don't recall

Across your collaborative research teams, how often have team discussions about the use of AI tools occurred <u>before</u> beginning the work?

- Always
- Sometimes
- Never

Have you ever used an AI tool to support any aspect of your work on a collaborative research team?
This could include any use, such as drafting emails, summarizing papers, copyediting, brainstorming, etc. It does not have to be frequent or central to your work.

- Yes
- No
- Not sure

[*if yes to ever used an AI tool*]
Approximately how often do you use AI tools in your work on collaborative research teams?

- Daily
- Weekly
- Monthly
- Rarely (a few times per year or less)

[*if yes to ever used an AI tool*]
When did you first start using AI tools to support your research workflows or collaborations?

- Within the past 6 months
- 6 months to 1 year ago
- 1 to 2 years ago
- More than 2 years ago
- Not sure / Can't recall

[*if yes to ever used an AI tool*]
Across any of your research collaborations, for which of the following research tasks have AI tools been used to support the work?
Check all that apply.

- Designing studies or planning research projects
- Conducting literature reviews or synthesizing background materials
- Developing marketing or outreach materials (e.g., for participant recruitment)
- Drafting or editing regulatory documents (e.g., IRBs, study protocols, DSMB charters)
- Developing statistical analysis plans
- Generating or debugging code for statistical or computational analyses
- Interpreting outputs from statistical or computational analyses
- Running data analyses directly within an AI tool (e.g., uploading data into an AI interface)
- Drafting or editing dissemination materials (e.g., manuscripts, grant proposals, abstracts)
- Preparing visual materials (e.g., slide decks, figures, data visualizations)
- Other [*free text*]

[*if yes to ever used an AI tool*]
Across any of your research collaborations, for which of the following communication or coordination tasks have AI tools been used?
Check all that apply.

- Drafting or editing emails or messages
- Creating meeting agendas or planning documents
- Taking automated meeting notes (e.g., via transcription tools)
- Summarizing or outlining meeting notes or discussions
- Clarifying team roles or responsibilities (e.g., drafting charters, identifying expertise gaps)
- Mediating conflict or misunderstandings (e.g., drafting neutral responses, rephrasing feedback)
- Supporting onboarding of new team members (e.g., generating overviews, orientation materials)
- Other [*free text*]

[*if yes to ever used an AI tool*]
Which AI tools have you used to support your research workflows or collaborations?
Select all that apply.

- Canva with AI features
- ChatGPT (OpenAI)
- Elicit
- GitHub Copilot
- Google Gemini
- Grammarly
- Microsoft Copilot
- Otter.ai

- Perplexity
- Scite
- SciSpace (formerly Typeset.io)
- Other [*free text*]

<u>General Perspectives about AI in Collaborative Research</u>

Please indicate your level of agreement with the following statements about the use of AI tools in collaborative research.

Strongly Disagree / Disagree / Neutral / Agree / Strongly Agree

- Using AI tools reduces administrative and repetitive tasks, allowing teams to focus more on science.
- The integration of AI tools enhances the creativity and innovation of research teams.
- Overreliance on AI tools could undermine critical thinking within research teams.
- AI tools contribute to more equitable participation and voice among team members.
- AI tools help bridge communication gaps between members of different disciplines.
- AI tools have the potential to transform how research findings are shared with broader audiences.
- Training and support are necessary to maximize the benefits of AI tools in research collaborations.

How do you think AI tools impact disparities between well-resourced and less-resourced research teams?

- AI tools <u>reduce disparities</u> by providing more equitable access to resources and capabilities
- AI tools <u>increase disparities</u> by favoring groups with more access to technology and expertise
- AI tools have <u>no significant impact</u> on disparities
- I'm not sure / No opinion

What concerns do you have about using AI tools in collaborative research?
Select all that apply.

- Accuracy of outputs/misinformation
- Bias in AI models
- Data/privacy concerns
- Environmental sustainability
- Intellectual property/plagiarism concerns
- Job displacement
- Lack of transparency ("black box")
- Misuse or overreliance on AI
- Uncertainty about policies on AI use in collaborative research
- I have no concerns about using AI tools in collaborative research
- Other [*free text*]

Framework

Who should be responsible for deciding how AI tools are used (or not used) within a collaborative research team?
Please select the option that best reflects your perspective.

- The Principal Investigator (PI) or team leader should make the decision for the team.
- Each team member should decide independently how they use AI tools in their own work.
- The team should make this decision collectively, though consensus or group agreement.
- The institution or organization should establish guidelines that apply to all researchers in the organization.
- Other approach [*free text*]

Successful integration of AI tools into collaborative research workflows requires teams to discuss and develop clear internal policies about how AI will be used.

- Strongly disagree
- Disagree
- Neutral
- Agree
- Strongly agree
- I Don't Know

I would benefit from a validated framework or model for the responsible use of AI in collaborative research.

- Strongly disagree
- Disagree
- Neutral
- Agree
- Strongly agree
- I Don't Know

What topics or components would you find valuable in a framework or model covering the responsible use of AI tools in collaborative research?
Select all that apply.

- Guidelines for ethical and responsible AI use
- Recommendations on team communication and coordination around AI
- Guidelines on defining roles and responsibilities related to AI tool use
- Procedures for evaluating and selecting AI tools before adoption
- Strategies to address data privacy and security concerns
- Training resources that help team members learn how to effectively use AI tools
- Metrics or methods to assess AI's impact on team productivity and research outcomes
- Approaches for managing disagreements or challenges related to AI-generated results or content
- Other [*free text*]
- None of the above – I would not find such a framework valuable.

If you received training or guidance on responsibly using AI in collaborative research, what format(s) would you prefer?
Select all that apply.

- In-person workshops or seminars
- Online interactive courses or webinars
- Written guidelines or best practice documents
- One-on-one coaching or consultation
- Peer discussion groups or communities of practice
- Other [*free text*]

Open-Ended Reflection

These questions are optional. If you do not wish to provide reflections on these topics, please leave them blank and click "Submit."

Describe a time when AI improved a collaborative research project you were part of. [*free text*]

Describe a time when AI disrupted a collaborative research project you were part of. [*free text*]

If you have any other thoughts about the use of AI tools in collaborative research, please share them here. [*free text*]
